# GW4869 Depletes Macrophages and Increases Number of Extracellular Vesicles in Murine Peritoneal Cavity Fluid

**DOI:** 10.1002/jex2.70169

**Published:** 2026-08-28

**Authors:** Johann Mar Gudbergsson, Pedro Henrique Gobira, Rodrigo Grassi‐Oliveira, Anders Etzerodt

**Affiliations:** ^1^ Department of Biomedicine Aarhus University Aarhus Denmark; ^2^ Translational Neuropsychiatry Unit, Department of Clinical Medicine Aarhus University Aarhus Denmark; ^3^ Nanopsychiatry Lab, School of Medicine Pontifical Catholic University of Rio Grande do Sul (PUCRS) Porto Alegre Brazil

**Keywords:** ApoB, CD9, inflammation, inhibition, in vivo, exosome, lipoprotein particle, microvesicle, nano flow cytometry, nanoflow

## Abstract

Pharmacological strategies to modulate extracellular vesicle (EV) release in vivo are gaining traction, yet their broader effects on local immune microenvironments remain unclear. In this study, we assessed how repeated intraperitoneal (i.p.) dosing of the neutral sphingomyelinase inhibitor GW4869 reshapes the peritoneal cavity. GW4869 administration triggered a strong local inflammatory reaction, was accompanied by a selective depletion of resident peritoneal macrophages and was associated with higher EV counts 24 h after the final injection. Targeted macrophage depletion in the absence of GW4869 produced a comparable increase in EV levels, implicating shifts in cellular composition and inflammatory state as key drivers of EV accumulation. By applying single‐particle flow cytometry, we differentiated small EV subsets from ApoB+ lipoproteins and uncovered substantial heterogeneity within CD9+ particles, ruling out lipoprotein co‐detection as the main explanation for the elevated EV signal. Overall, our results highlight that GW4869 treatment can reshape the local immune landscape and that such context‐dependent changes must be considered when using this compound to infer EV biogenesis in vivo.

## 1 Introduction

Extracellular vesicles (EVs) are a collection of heterogeneous lipid bilayer vesicles, including late endosome‐derived exosomes and plasma membrane‐derived microvesicles. Exosomes are usually described as the smallest EVs, ranging from 30 to 200 nm, whereas microvesicles range from 100 to 1000 nm. Subclasses of EVs are in theory defined by their pathway of biogenesis but often in practice defined by size and/or density, such as large EVs being membrane‐derived microvesicles and small EVs being exosomes. The link between EV size and biogenesis is not straightforward, if it exists at all, and consequently we will refer to them as small and large EVs. EVs have been shown to play key roles in health and disease by affecting neighbouring or distant cells with their cargo, and inhibition of EV release has been crucial for understanding their role in cell‐to‐cell communication (van Niel et al. 2022). Several small‐molecule inhibitors have been shown to affect EV secretion, including calpeptin, manumycin A, imipramine and the neutral sphingomyelinase 2 (nSMase2) inhibitor GW4869 (Catalano and O'Driscoll 2020). nSMase2 generates ceramide that is important for intraluminal vesicle formation and exosome secretion, and GW4869 has been instrumental for elucidating role of ceramide in the exosomal pathway (Trajkovic et al. 2008). Consequently, GW4869 has become one of the most widely used EV inhibitors and has helped demonstrate how EVs contribute to diverse processes, including viral transmission (Sultana et al. 2024), cell motility (Pericoli et al. 2023), education of macrophages (Peng et al. 2022), and response to chemotherapy (Irep et al. 2024). Although GW4869 is commonly described as well tolerated following intraperitoneal (i.p.) administration in mice, systematic assessments of local immune or cellular effects are limited, and reports of its ability to reduce EV release in distant tissues, such as the brain, have relied largely on indirect or heterogeneous measurements (Essandoh et al. 2015; Habibi et al. 2022; Mowry et al. 2023; Dinkins et al. 2014; Tabatadze et al. 2010). Only two studies have also investigated peritoneal cells or EVs after i.p. injection of GW4869 without mentioning adverse effects on the cellular compartments, indicating that the inhibitor is well‐tolerated locally as well (Bhattacharya et al. 2023; Wang et al. 2023). To directly test whether GW4869 reduces EV abundance in vivo, we specifically evaluated its effects on EV levels and cellular composition within the peritoneal cavity. Here, we found that repeated intraperitoneal injections of GW4869 induced local inflammation and selective depletion of resident macrophages. Unexpectedly, rather than reducing EV abundance, we observed a significant increase in peritoneal EV numbers 24 h after the final injection. Independent macrophage depletion reproduced both the rise in EVs and the influx of inflammatory cells, indicating that changes in the cellular milieu, rather than direct inhibition of EV biogenesis, largely explain this effect. Together, these results demonstrate that GW4869 markedly perturbs local immune homeostasis in vivo, which can secondarily alter EV levels and complicates its use as a selective EV inhibitor.

## 2 Materials and Methods

### 2.1 Animals and Housing

Male C57BL/6J mice (8 weeks old; Janvier Labs, France) were group‐housed (five per cage) under controlled environmental conditions (22 ± 2°C; 60 ± 5% relative humidity) with ad libitum access to food and water. Animals were maintained on a 12 h light/dark cycle (lights on at 06:00 h) and provided with environmental enrichment, including a stainless‐steel tunnel shelter, nesting material, and a wooden stick, throughout the study. The in vivo ShredR model was performed using Cas9‐EGFP^fl/fl^ (B6J.129(B6N)‐Gt(ROSA)26Sor<tm1(CAG‐cas9*,‐EGFP)Fezh>/J) mice bred in‐house. These mice were crossed with Csf1r^Cre/+^ (C57BL/6‐Tg(Csf1r‐cre)1Mnz/J) mice to generate animals with Csf1r‐dependent Cas9 expression. All experimental procedures were approved by the Danish National Committee for Ethics in Animal Experimentation (protocol 2023‐15‐0201‐01528 for GW4869 studies and 2021‐15‐0201‐01083 for the ShredR‐LNP study) and were conducted in accordance with the European Community Council Directive 2010/63/EU.

### 2.2 Drug and Treatment Protocol

Prior to the start of drug administration, animals were acclimatized to the housing facility for 2 weeks. The neutral sphingomyelinase inhibitor GW4869 (TargetMol Chemicals Inc, USA) was prepared as an 8 mg/mL stock solution in dimethyl sulfoxide (DMSO) and freshly diluted in sterile saline to a final DMSO concentration of 2.5% immediately before use. Mice received daily i.p. injections of GW4869 (2.5 mg/kg) or vehicle (saline + 2.5% DMSO) for 14 consecutive days. This dosing regimen was selected based on previous studies demonstrating effective in vivo inhibition of neutral sphingomyelinase activity and EV release following chronic systemic administration of GW4869 (Dinkins et al. 2014; Tabatadze et al. 2010; Asai et al. 2015). Animals were randomly assigned to one of two treatment groups (vehicle or GW4869) and sacrificed by decapitation either 2 h or 24 h after the final injection. Mice were acclimatized for 2 weeks before receiving daily i.p. (i.p.) injections of GW4869 (2.5 mg/kg) or vehicle for 14 consecutive days. Animals were sacrificed either 2 h or 24 h after the final injection to assess potential temporal effects of nSMase inhibition. Peritoneal lavage samples were collected immediately after sacrifice.

### 2.3 Macrophage Depletion Using ShredR Lipid Nanoparticles

Lipid nanoparticles (LNPs) loaded with ShredR sgRNA were prepared as previously described (see Table 1 for lipid formulation and sgRNA sequences) (Rasmussen et al. 2025). Briefly, RNA dissolved in the aqueous phase (100 µM sodium acetate buffer, pH 4.5; Thermo Scientific) was combined with the lipid phase at a flow rate ratio of 4:1 (aqueous to lipid), maintaining a total flow rate of 5 mL/min using the NanoAssemblr platform. The resulting LNPs were subsequently dialyzed twice overnight against 1xPBS (Cytiva) using 14 kDa MWCO dialysis tubing (Spectra/Por 4). To concentrate the preparation, the dialysis membranes were covered with polyethylene glycol (PEG, Mw 35,000; Sigma–Aldrich) for 10–20 min at room temperature. Afterward, the LNPs were passed through a 0.2 µm cellulose acetate sterile filter (Avantec). Lipid content was quantified by high‐performance liquid chromatography (HPLC) using a Dionex Ultimate 3000 system (Thermo Fisher Scientific) equipped with a Nucleosil 100‐3C18 column. RNA concentration was assessed using the modified Quant‐iT RiboGreen RNA assay (Thermo Scientific #R11490) following the Precision NanoSystems protocol. For storage, LNPs were frozen at −70°C in the presence of 5% sucrose (Merck) as a cryoprotectant to ensure RNA stability. For in vivo use, LNPs were diluted in sterile PBS and 250 ng LNP (based on RNA concentration) was injected i.p. in 0.2 mL into CD115^Cre^ × Cas9‐GFP^fl/fl^ and control mice. Peritoneal cells and fluid were harvested 24 h later as described below.

**TABLE 1:** LNP formulation and sgRNA sequences.

LNP formulation		
Reagent	Lipid ratio	Manufacturer
SM‐102	50	BOC Sciences
DSPC	10	Cayman
Cholesterol	39	Sigma Aldrich
DMG‐PEG(2000)	1	Cayman
**sgRNA**	**Sequence**	
ShredR	TGCCTCTGCCTCCCAAGTGC	
Scrambled control	GCTTAGTTACGCGTGGACGA	

### 2.4 Extraction of Peritoneal Cells and Fluid

After decapitation, mice were injected i.p. with 5 mL of PBS + 2 mM EDTA (Invitrogen, #15575020), and the abdominal region was massaged for 5–10 s prior to extraction of the fluid, as previously described (Gudbergsson et al. 2025). To extract the fluid, a small incision was made in the skin and blunt dissection done to expose the outer muscle layer. The lateral skin on the right side of the mouse was left attached to allow for stretching of the abdominal muscle layer and displace the abdominal organs with a finger to create an i.p. space for fluid aspiration. A 25G needle was inserted into the peritoneal cavity rostrally pointing caudally and fluid was slowly aspired. Approximately 4.5 mL could be recovered from each mouse. The peritoneal fluid was then centrifuged at 400×*g* for 5 min at 4°C to pellet cells and cells were resuspended in FACS buffer (PBS, 2.5 % FBS, 1 mM EDTA, 0.1 % NaN3) for further analyses. The supernatant was transferred to a new tube and centrifuged for 15 min. at 2000×*g* at 4°C to pellet cell debris. The resulting supernatant was aliquoted, frozen and stored at ‐20°C until analysis.

### 2.5 Spectral Flow Cytometry

Peritoneal cells were counted on a NucleoCounter NC‐3000 using A8 NC‐slides and viability solution 13 (ChemoMetec). Stain mix was prepared using FACS buffer with mouse Fc‐block (clone 2.4G1), brilliant stain buffer (#659611, BD Horizon) and oligo block (Kristensen et al. 2021), and antibodies (see antibody panel and dilutions in Table 2). 1 million cells were stained at RT for 30 min, resuspended in FACS buffer + viability dye, and 300–400.000 events were recorded on a SONY ID7000 spectral flow cytometer using standardized mode. Reference spectra were made from antibody single stains using UltraComp eBeads Plus compensation beads (01‐3333‐42, Invitrogen) and spectra for viability dye was made using ViaComp beads (SSB‐07‐A, Slingshot Biosciences). Individual spectra were loaded to the sample groups and unmixing was done using the SONY ID7000 Software. After unmixing, sample analyses were done using FlowJo 10.10.0 (BD Life Sciences), and graphs and statistics were done with GraphPad Prism 10 (Dotmatics). Gating strategy for cells can be seen for GW4869 and vehicle treatments in Figure S1A.

**TABLE 2:** Spectral flow panel.

Primary excitation laser	Antigen	Clone	Catalogue	Vendor	Fluorochrome	Dilution
**UV 355nm**	NK1.1	PK136	564144	BD Horizon	BUV395	200
	CD102	3C4(mIC2/4)	751392	BD Optibuild	BUV615	400
	Tim4	RMT4‐54	749134	BD Optibuild	BUV737	400
	CD19	1D3	568287	BD Horizon	BUV805	400
**Violet 405nm**	SYTOX Blue		S34857	Invitrogen		10000
	F4/80	T45‐2342	743282	BD Bioscience	BV650	400
	CD115	AFS98	135515	BioLegend	BV711	400
	Siglec F	E50‐2440	740956	BD Optibuild	BV786	400
**Blue 488nm**	Lyve1	ALY7	53‐0443‐82	Invitrogen	AF488	800
	CD45	30‐F11	569257	BD Horizon	RB545	400
	Ly6C	AL‐21	571103	BD Horizon	RB613	400
	CCR2	475301	757170	BD Optibuild	RB744	400
**Yellow 561nm**	CD9	MZ3	124805	BioLegend	PE	4000
	CD3e	145‐2C11	MCA2690C	BioRad	PE‐Cy5	400
	CD226	10E5	567651	BD Pharmingen	PE‐Cy7	800
**Red 637nm**	FOLR2	10/FR2	153305	BioLegend	APC	800
	Ly6G	1A8	560600	BD Pharmingen	APC‐Cy7	200
	CD11b	M1/70	101287	BioLegend	APC‐Fire/810	800

### 2.6 Nano Flow Cytometry

All nano flow cytometry experiments were performed on a CytoFLEX Nano (Beckman Coulter), as previously described (Gudbergsson et al. 2025). We did not enrich for EVs and kept samples untouched apart from the dilution during extraction of peritoneal fluid. 50 uL of sample was added to a 96‐well plate and stained with 50 uL anti‐CD9‐AF647 antibody (clone MZ3, #124809, BioLegend) and/or anti‐ApoB‐CoraLite‐Plus‐488 (clone 240884H4, # CL488‐84047, Proteintech) diluted to 1 µg/mL in PBS, which was added on top and mixed. Antibody titrations are shown in Figure S3F. Prior to making antibody stain mix, all antibody vials were centrifuged at 16,000 × g for 5 min to avoid/reduce antibody aggregates. Plate was incubated overnight at 4°C with antibody. The next day, samples were stained at 1X concentration with a mix of the membrane dyes ExoBrite 410/450 CTB (#30111‐T, Biotium) and ExoBrite 515/540 True EV membrane stain (#30129‐T, Biotium) or 10 nM MemGlow640 (#MG04, Cytoskeleton, Inc) which was diluted in PBS and 50 uL added to the samples and incubated 1–2 h at RT. Just before analysis samples were diluted in PBS to a final dilution of 1:200 and run on the CytoFLEX Nano. A vehicle (PBS) control sample was run to set the vSSC1 threshold which in our setup was set at 300 in all experiments. Scatter detectors gains were kept according to QC values, but relevant fluorescent detectors were maxed to 3000. Single stain and unstained controls were run to setup compensation. PBS + stain control was run as a background control and for all quantifications the background values were subtracted using the PBS + stain control. A detergent control was used where 2% Triton X‐100 (CAT) was prepared and sterile filtered, and added 1:1 to an already analysed sample and incubated for 30 min at RT. The sample was then analysed to show that the particles that fall into our EV gate are in fact sensitive to detergents. Scatter calibration was done as previously described using FCMPASS, with sEVs being calibrated according to the “Average EV RI” and the ApoB single positive population according to “Enveloped Virus” with a RI of 1.456 which fitted best with typical RI for vLDL and LDL between 1.44 and 1.46 (Gudbergsson et al. 2025; Welsh et al. 2020; Welsh and Jones 2020). We have compiled representative controls in Figure S2. Compensation and data analysis was done in CytExpert Nano (Beckman Coulter) and FlowJo v10.10.0 (BD Life Sciences) and quantitative graphs and statistical analyses were created in GraphPad Prism 10 (Dotmatics).

## 3 Results

### 3.1 GW4869 EV Inhibitor Depletes Macrophages and Causes Inflammation in the Mouse Peritoneal Cavity

**FIGURE 1:**
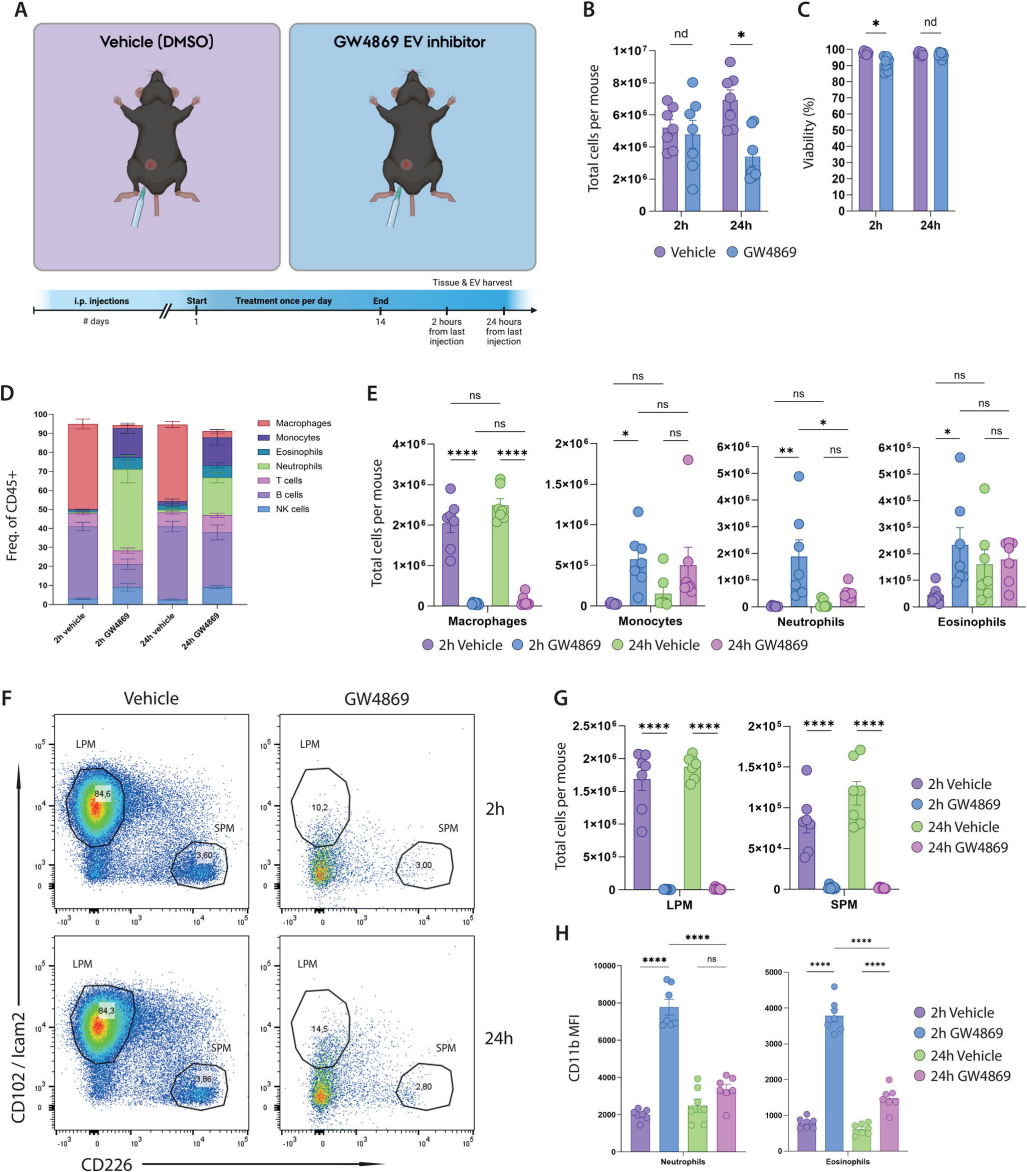
GW4869 depletes peritoneal macrophages and induces local inflammation. (A) Schematic overview of treatment schedule. (B) Total peritoneal cells per mouse (*p* = 0.00151). (C) Viability of cells (*p* = 0.00154). (D) Stacked bar charts of cell types represented as frequency of CD45^+^ cells. (E) Bar charts of total number of macrophages (*p* = <0.0001), monocytes (*p* = 0.0385), neutrophils (*p* = 0.0017, *p* = 0.0282) and eosinophils (*p* = 0.036). (F) Representative bivariate plots of LPM and SPM populations. (G) Bar chart showing total LPMs (*p* = <0.0001) and SPMs (*p* = <0.0001) per mouse. (H) CD11b MFI of neutrophils (*p* = <0.0001) and eosinophils (*p* = <0.0001). *n* = 7.

In an attempt to inhibit EV release from mouse peritoneal cells, we treated mice i.p. for 14 consecutive days with the nSMase2 inhibitor GW4869 or vehicle (Figure 1A). To analyse the effect on EV release and cellular dynamics we harvested peritoneal cells and EVs 2 and 24 h after the last injection. The two time points were selected to capture temporal changes occurring within the first 2 h after treatment compared with those present at 24 h. Surprisingly, we observed a specific reduction of total cells in GW4869 treated mice 24 h after last injection compared to vehicle control. In contrast, there was no major difference in cell viability, nor did we observe any difference in cell numbers 2 h after last (Figure 1B, C). This observation warranted a deeper phenotypic analysis of the peritoneal cells after GW4869 treatment using spectral flow cytometry to detect any changes in cell populations. Strikingly, we observed that GW4869 treatment specifically depleted peritoneal cavity of macrophages at both timepoints (Figures 1D–E and S1B). Further analysis showed that both large peritoneal macrophages (LPM) and small peritoneal macrophages (SPM) were depleted after treatment, as remaining F4/80+CD11b+ macrophages did not express canonical LPM markers Icam2/CD102 or the SPM (CD226) markers (Figure 1F, G). Simultaneously, a high neutrophil and eosinophil influx was seen 2 h after treatment, along with a significant increase in CD11b MFI indicating an inflammatory environment (Figure 1E, H). The number of neutrophils and eosinophils and their respective CD11b MFI was reduced after 24 h, indicating a strong and short‐lived response to GW4869 (Figure 1E, H). In summary, we found that the nSMase2 inhibitor GW4869 eliminates the macrophage compartment in the peritoneal fluid with the dose applied and creates an inflammatory environment with activated neutrophils and eosinophils.

### 3.2 CD9 is a Robust sEV Marker and Present in Murine Peritoneal Cavity Cells and Peritoneal Fluid EVs

**FIGURE 2:**
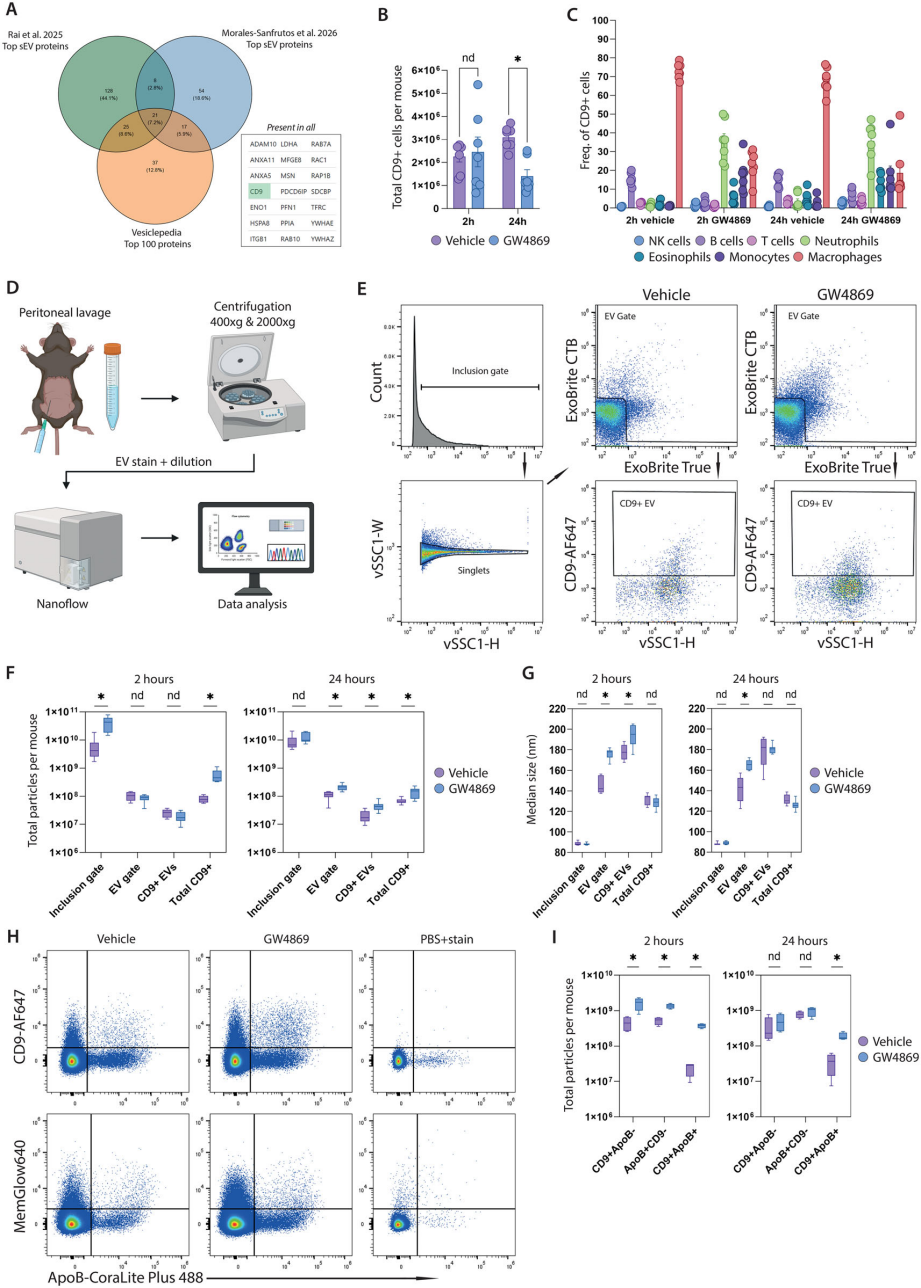
GW4869 increases number of total peritoneal EVs. (A) Venn‐diagram showing the overlapping proteomic signature enriched in sEVs. (B) Total CD9^+^ cells per mouse (*p* = 0.00026). (C) Bar graph showing the change of cell distribution within CD9^+^ cells. (D) Brief schematic overview of sample workflow. (E) Gating strategy for nano flow cytometry analyses peritoneal particles and EVs. (F) Boxplots (min–max) showing total particles per mouse for inclusion gate, EV gate, CD9^+^ EVs and total CD9^+^ at 2 and 24 h after last GW4869 treatment. 2 h inclusion gate (*p* = 0.00142), 2 h total CD9^+^ (*p* = 0.001), 24 h EV gate (*p* = 0.00338), 24 h CD9^+^ EVs (*p* = 0.01146), 24 h total CD9^+^ (*p* = 0.0082). *n* = 7. (G) Median size in nm derived from size calibration using FCMPASS within inclusion gate, EV gate, CD9^+^ EVs and total CD9^+^ at 2 and 24 h after last GW4869 treatment. 2 h EV gate (*p* = 0.000002), 2 h CD9^+^ EVs (*p* = 0.008), 24 h EV gate (*p* = 0.00062). *n* = 7. (H) Bivariate plots of Vehicle, GW4869 and PBS + stain with ApoB co‐stained with either CD9 or MemGlow640. Concatenated samples (*n* = 4) were used. (I) Total particles per mouse represented as boxplots (min–max) for CD9^+^ApoB^−^, ApoB^+^CD9^−^, and CD9^+^ApoB^+^ at 2 and 24 h after last GW4869 treatment. 2 h CD9^+^ApoB^−^ (*p* = 0.0118), 2 h ApoB^+^CD9^−^ (*p* = 0.00048), 2 h CD9^+^ApoB^+^ (*p* = 0.000013), 24 h CD9^+^ApoB^+^ (*p* = 0.00113). *n* = 4.

After assessing the consequence of GW4869 treatment on the cellular compartment, we wanted to examine the effects of the nSMase2 inhibitor on particles and EV release in the peritoneal fluid using nano flow cytometry. First, we wanted to select an appropriate sEV marker that could reflect the cellular environment in the peritoneal cavity. We compared the top 100 protein markers from Vesiclepedia to two recently published studies that comprehensively investigated the defining markers of sEVs from plasma and cell lines and found that CD9 was represented in all three datasets (Figure 2A) (Chitti et al. 2024; Rai et al. 2025; Morales‐Sanfrutos et al. 2026). From our spectral flow cytometry experiments on peritoneal cells, we could show that approximately half of the cells in the peritoneal cavity expressed CD9; in vehicle‐treated mice this was mainly due to macrophages, whereas in GW4869‐treated mice CD9 expression was instead largely associated with neutrophils, eosinophils, and monocytes (Figure 2B, C). Further analyses showed that all neutrophils, eosinophils, and macrophages were CD9^+^, and that CD9 MFI in macrophages decreased in GW4869‐treated mice, whereas it increased to varying degrees in monocytes and eosinophils (Figure S3A, B). We have previously described CD9^+^ EVs in cell‐free peritoneal fluid from steady‐state mice, so to test for CD9^+^ EVs in this experiment we titrated the antibody on a pooled sample and analyzed them by nano‐flow cytometry using a CytoFLEX Nano (Beckman Coulter, Figure S3F) (Gudbergsson et al. 2025).

### 3.3 GW4869 Treatment Results in Increased Number and Size of sEVs in Murine Peritoneal Fluid

After establishing CD9 as a robust sEV marker and the presence of CD9 in both cells and EVs in the peritoneal fluid, we performed nano–flow cytometry on cell‐free peritoneal fluid collected 2 and 24 h after the final treatment (Figure 2D). All samples were acquired on the same day. An inclusion gate was first defined based on the dilution buffer, followed by a singlet gate to avoid particle swarming. To specifically analyse single EVs, samples were stained with a lipophilic dye (ExoBrite TrueEV Membrane Stain), a cholera toxin B conjugate (ExoBrite CTB), and an anti‐CD9 antibody (Figure 2E). We quantified particles within the inclusion gate, the EV gate, CD9^+^ EVs, and total CD9^+^, and observed a marked increase in events within the inclusion gate in GW4869‐treated mice at 2 h, which returned to baseline by 24 h (Figure 2F). Surprisingly, the total number of EVs was comparable between groups at 2 h but was increased approximately two‐fold at 24 h in GW4869‐treated mice (Figure 2F, Figure S3C–E). This increase was mirrored in CD9^+^ EVs at 24 h and was even more pronounced for total CD9^+^ particles at both time points (Figure 2F, Figure S3E). We also assessed particle size and detected an increased median diameter of EV‐gate particles in treated animals at both 2 h (145.8 nm vs. 175.9 nm) and 24 h (142 nm vs. 165 nm), and of CD9^+^ EVs at 2 h (177.6 nm vs. 193.5 nm), whereas no size changes were observed for particles in the inclusion gate, total CD9^+^ particles at either time point, or CD9^+^ EVs at 24 h (Figure 2G). In summary, we find that the EV inhibitory effects of GW4869 seem to be overshadowed by an induced inflammation in the mouse peritoneal cavity at the dose used here, and instead highly increases the presence of particles and EVs, likely as a consequence of macrophage depletion and influx of other activated immune cells.

### 3.4 GW4869 Treatment Increases Association Between sEVs and ApoB^+^ Lipoprotein Particles

Since the size and concentration of CD9^+^ EVs and total CD9^+^ particles differed so much and that the overlap between CD9 and the ExoBrite membrane stains was relatively modest (Figure S4A), we wondered whether ApoB^+^ lipoprotein particles (LDL, vLDL) could ‘contaminate’ the EV pool and perhaps even associate with the EVs to potentially inflate their estimated size. Here, we saw that a majority of ApoB^+^ particles did not stain for lipophilic dye (MemGlow640) or CD9, but double positives were present (Figure 2H). We plotted the particle concentrations for CD9^+^ApoB^−^, ApoB^+^CD9^−^ and CD9^+^ApoB^+^, and found all particle populations being increased in GW4869‐treated mice at 2 h, but only the CD9^+^ApoB^+^ double positive particles were significantly increased at 24 h (Figure 2I). The number of CD9^+^ApoB^−^ EVs and ApoB^+^CD9^−^ lipoprotein particles were quite similar within each treatment group. Looking at the size, however, we could see a substantial difference between CD9^+^ApoB^−^ and MG640^+^ApoB^−^ (88.6 nm vs. 129 nm for Vehicle, 100.5 nm vs. 153.7 nm for GW4869) and between CD9^+^ApoB^+^ and MG640^+^ApoB^+^ (110 nm vs. 155.6 for Vehicle, 125.3 nm vs. 173.3 nm for GW4869), whereas the ApoB^+^CD9^−^ and ApoB^+^MG640^+^ were highly similar (58 nm vs. 58.2 nm for Vehicle, 63.7 nm vs. 66.4 nm for GW4869) (Figure S4B). This could indicate that the MG640 predominantly associated with the larger fraction of the sEVs and the EV‐lipoprotein ‘aggregates’ whereas the ExoBrites showed a similar pattern as the total CD9^+^ (Figure S4C, D).

### 3.5 Specific Depletion of Peritoneal Macrophages Increases the Number of EVs

**FIGURE 3:**
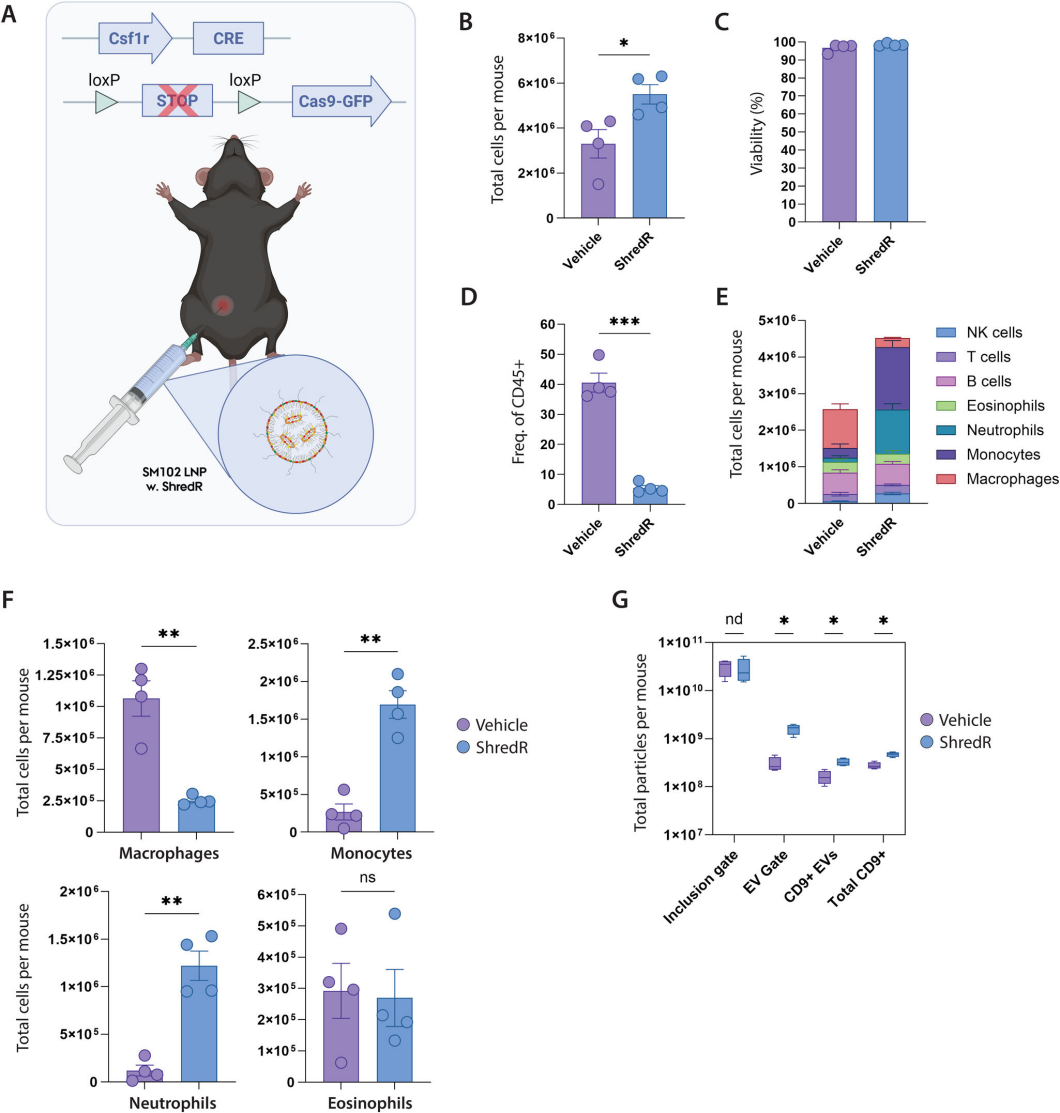
Macrophage depletion increases number of total peritoneal EVs. (A) Illustration of mouse model and treatment applied. (B) Total peritoneal cells per mouse (*p* = 0.0332). (C) Viability of cells. (D) Bar chart showing macrophage frequency of CD45^+^ cells (*p* = 0.0009). (E) Stacked bar charts of cell types represented as frequency of CD45^+^ cells and as total cells per mouse. (F) Bar charts representing total macrophages (*p* = 0.0096), monocytes (*p* = 0.0013), neutrophils (*p* = 0.0031), and eosinophils per mouse. (G) Box plot (min‐max) of total particles per mouse for inclusion gate, EV gate (*p* = 0.005), CD9^+^ EVs (*p* = 0.0048), and Total CD9^+^ (*p* = 0.0016). *n* = 4.

Since GW4869 treatment resulted in loss of peritoneal macrophages, subsequent inflammation and increase in EVs numbers, we next wanted to test if the effects were specifically due to depletion of the peritoneal macrophages. To this end we specifically depleted macrophages by breeding Csf1r‐Cre mice with Cas9‐egfp floxed mice to generate Cas9‐expressing macrophages in the peritoneal environment and delivered SM102 lipid nanoparticles containing our cell‐depleting ShredR sgRNAs (Figure 3A), as previously described (Rasmussen et al. 2025). Although ShredR treatment led to an increase in total peritoneal cells with no difference in cell viability (Figure 3B, C), flow cytometric analysis confirmed an efficient depletion of macrophages after 24 h (Figure 3D). As hypothesized, the depletion of LPMs and SPMs was accompanied by an influx of monocytes and neutrophils as previously observed with GW4869 treatment (Figure 3E, F). Interestingly, although total number of particles present in the peritoneal fluid was not different between the treatments, ShredR treatment led to a specific increase in total EVs, CD9^+^ EVs and total CD9^+^, similar to what we observed for GW4869 treatment (Figure 3G–I). These results indicate that a depletion of macrophages from the peritoneal cavity fluid results in an inflammatory environment which is accompanied by an increased number of EVs.

## 4 Discussion

The effects of GW4869 on immune cell subsets in vivo has not been extensively characterized. In our study, examining its impact on mouse peritoneal cells, we observed an almost complete depletion of macrophages from the peritoneal fluid. This depletion resulted in local inflammation with increased neutrophil and eosinophil influx, and instead of a reduction of EV abundance, we saw a significant increase in total EVs, CD9^+^ EVs and total CD9^+^ particles 24 h after the final treatment. We verified this finding by specifically depleting peritoneal macrophages, as previously described (Rasmussen et al. 2025), which indicates that depletion of peritoneal macrophages results in local inflammation which could overshadow the EV inhibitory effects of GW4869.

The impact of GW4869 on EV numbers has largely been ascribed to reduced EV release, rather than to potential cellular consequences such as depletion of specific cell populations or alterations in cellular lipid composition (Lin et al. 2019; Martín‐Acosta and Xiao 2020; Thayyullathil et al. 2021). Nevertheless, inhibition of nSMase2 has previously been reported to perturb fundamental cellular processes, including TNF signalling and autophagy‐related pathways (Luberto et al. 2002; Back et al. 2018). Because ceramide is a central mediator of inflammatory signalling and contributes to inflammasome assembly and IL‐1β secretion, pharmacological inhibition of nSMase2 is likely to affect immune cells such as macrophages beyond merely reducing EV release (Atreya and Neurath 2024; Scheiblich et al. 2017; York et al. 2024). Consistent with this, GW4869 has been shown to exert anti‐inflammatory effects in several disease models, with evidence for direct actions on macrophages (Al‐Rashed et al. 2020; Lallemand et al. 2018; Kumar et al. 2019). Thus, in our study, the inflammation is likely to be indirectly caused by the GW4869‐mediated depletion of macrophages and not bona fide induction of inflammatory pathways, since specific depletion of the macrophage compartment showed similar effects. It is important to notice that depletion of macrophages from the peritoneal fluid does not necessarily imply cell death, as macrophages can leave the fluid and migrate to relevant tissue upon activation often referred to as the ‘macrophage disappearance reaction’ (Louwe et al. 2021). Nonetheless, inflammation has generally been reported to enhance EV release from cultured cells and to associate with higher circulating EV levels in adults with obesity‐related adipose tissue inflammation (Yang et al. 2018; Matilainen et al. 2024; Wang et al. 2017). The inclusion of two time points (2 and 24 h) allowed for the assessment of potential temporal effects of nSMase inhibition, controlling for confounding influences related to the prolonged 24 h drug‐free interval and potential peak enzymatic activity of neutral sphingomyelinase. At the 2 h time point we saw no difference in total EVs as determined by the staining with the two ExoBrite membrane stains, but a vast difference in total particle numbers and total CD9^+^ particles. At 24 h we saw an increase in EV gate, CD9^+^ EVs and total CD9^+^ particles. This might indicate that GW4869 may, to some extent, have attenuated inflammatory EV production by infiltrating cells in our experiments. This time‐dependent pattern could reflect declining GW4869 activity alongside cellular turnover. In parallel, given the established role of macrophages in EV clearance, loss of these cells may further contribute to EV accumulation in the peritoneal fluid (Tang et al. 2022; Imai et al. 2015).

An increase in EVs after GW4869 treatment has not, to the best of our knowledge, been reported before, but previous studies showed that GW4869 treatment could specifically reduce exosome release and increase microvesicle/apoptotic vesicle release (Menck et al. 2017; Risner et al. 2023). However, this conclusion is only based on increased number of particles (analysed by NTA) that precipitate at medium‐speed centrifugation (10–14,000×*g*) after GW4869 treatment compared to high‐speed (>100,000×*g*), and such analysis cannot properly distinguish between exosomes and microvesicles due to their overlap in size and density (Catalano and O'Driscoll 2020; Menck et al. 2017; Welsh et al. 2024). Interestingly, one of the studies showing inhibitory effect of GW4869 on EV release also reported an increase in apoptotic‐related Annexin A5 expression in the 10,000×*g* fraction, indicating a decrease in cellular health after GW4869 treatment (Risner et al. 2023). We could only find one study examining the peritoneal microenvironment following GW4869 treatment which reported no apparent adverse effects on peritoneal cells but did observe a reduction in EVs at a dose of 2.5 mg/kg, similar to the dose used here (Bhattacharya et al. 2023). Notably, this study employed a single GW4869 injection, whereas our study used a 14‐day treatment regimen, which may differentially affect macrophages. In addition, the study used a Zymosan A–induced sterile peritonitis model, which has been reported to deplete macrophages from the peritoneal cavity and to increase sphingolipid synthesis (Louwe et al. 2021; Fujieda et al. 2013; Pierron et al. 2023). Whether pre‐depletion of large peritoneal macrophages by sterile peritonitis before GW4869 injection results in fewer effects on cellular function is not known. For future in vivo studies using GW4869, dose‐titrations would be necessary to decrease any potential adverse effects in the local cellular environment.

Flow cytometry‐based EV analysis has grown steadily over recent years, with single‐EV phenotyping becoming increasingly important for advancing EV research. A major limitation has been the scarcity of flow cytometers specifically optimized for small‐particle detection with sufficient sensitivity for reproducible sEV phenotyping. Recently, several dedicated nanoscale instruments have been introduced, including the CytoFLEX Nano (Beckman Coulter), whose performance in EV analysis we have previously evaluated and validated (Gudbergsson et al. 2025). Instead of relying on bulk measurements of membrane stains, CD9 and ApoB, we were able to resolve their differential expression at the single‐particle level. Here, we define EVs as particles positive for either of the two ExoBrite membrane stains: ExoBrite CTB, which binds GM1 gangliosides, and ExoBrite True EV Membrane stain, which labels the lipid bilayer. We then distinguish two CD9^+^ populations: CD9^+^ EVs, which are positive for at least one membrane stain, and the total CD9^+^ population, which comprises all CD9^+^ particles (Total CD9^+^). Although, in principle, the Total CD9^+^ population could be considered EVs, the differential membrane staining observed among CD9^+^ particles led us to retain both terms to highlight a potential distinction between these subsets. To elucidate why not all CD9^+^ particles were positive for either of the membrane stains, we wanted to see whether CD9 was associated with ApoB^+^ lipoprotein particles and how both CD9 and ApoB overlapped with lipophilic dyes. To our surprise, we saw that the majority of ApoB^+^ lipoprotein particles were both negative for CD9 and lipophilic dye in contrast to what has previously been reported (Chen et al. 2023; Brealey et al. 2024). However, a minority of the ApoB^+^ lipoprotein particles did co‐stain with both lipophilic dye and CD9. Recently, Morales‐Sanfrutos et al. defined a reference proteome for sEVs from numerous cancer cell lines and biofluids and generated a top100 sEV‐associated protein list where ApoB was included (Morales‐Sanfrutos et al. 2026). This could indicate an association between EVs and ApoB^+^ lipoprotein particles, perhaps by interaction in the EV protein corona as has previously been suggested (Ripoll et al. 2026; Tóth et al. 2021). This was consistent with the double‑positive particles being larger than each singly labelled population. A similar tendency was observed when co‑staining with ApoB and the lipophilic dye MemGlow640. In these experiments, MemGlow640 additionally showed a clear preference for larger EVs compared with the ExoBrite labels, indicating differences in how the membrane dyes interact with EV membranes. Together, this shows that the increase in the different sEV populations in GW4869‐treated mice was not due to co‐staining of ApoB^+^ lipoprotein particles.

The observations reported in this study are primarily descriptive, and we fully acknowledge that we do not yet provide mechanistic insight into why peritoneal macrophages are depleted, why EV numbers increase, or from which cellular sources these EVs arise. Clarifying these points will require dedicated future work, including experiments that dissect the upstream signals driving macrophage loss, the pathways governing EV biogenesis under these conditions, and the relative contribution of distinct peritoneal cell types to the EV pool. Such studies, potentially combining lineage tracing, cell type‐specific EV labelling, and functional perturbation of candidate pathways, lie beyond the scope of the present manuscript but represent an important next step toward understanding the biological significance of the phenomena we describe.

### 4.1 Concluding Remarks

In conclusion, repeated intraperitoneal administration of GW4869 was associated with local inflammation, selective loss of resident peritoneal macrophages, and an unexpected increase in EV abundance 24 h after the final injection. Similar changes were observed after independent macrophage depletion experiments which support the notion that alterations in cellular composition and inflammatory status of the peritoneal cavity, rather than direct inhibition of EV biogenesis alone, might contribute to the elevated EV levels. We show that single‐particle flow cytometry enables robust discrimination of sEV subpopulations, revealing heterogeneity within CD9+ particles and minimal overlap with ApoB+ lipoprotein particles, thereby confirming that observed increases in sEVs are not driven by lipoprotein contamination. Together, these findings suggest that secondary immunological changes and tissue context should be carefully considered when employing GW4869 as a pharmacological EV inhibitor and when interpreting in vivo EV measurements.

## Author Contributions

**Johann Mar Gudbergsson**: conceptualization, investigation, methodology, data curation, validation, visualization, writing – original draft, writing – review and editing, formal analysis, project administration. **Pedro Henrique Gobira**: methodology, investigation, validation, writing – review and editing. **Rodrigo Grassi‐oliveira**: funding acquisition, writing – review and editing, supervision, resources. **Anders Etzerodt**: conceptualization, writing – review and editing, funding acquisition, supervision, resources.

## Conflicts of Interest

The authors declare no potential conflicts of interest.

## Supporting information




**Figure S1**: A) Gating strategy used for spectral flow analysis of cells. Plots are representative for each treatment group. B) Stacked bar charts of cell types represented as total cells per mouse.
**Figure S2**: Nano flow cytometry controls. A) Representative controls for EV staining using ExoBrite CTB and ExoBrite True EV Membrane stain. B) Representative controls for CD9‐AF647 staining.
**Figure S3**: A) Percentage of CD9^+^ cells within each defined cell type. B) Bar charts displaying CD9 MFI for macrophages (p = 0.0084, p = 0.0011), monocytes (p = 0.0242), neutrophils, and eosinophils (p = 0.0344, p = 0.0063). C) Repeat experiment of GW4869 i.p. 24 h time point; total peritoneal cells per mouse (p = 0.0102). n = 7–8. D) Viability of cells. E) Box plot (min‐max) of total particles per mouse of inclusion gate, EV gate, CD9^+^ EVs (p = 0.0011), Total CD9^+^ (p = 0.0194). F) Titration of CD9‐AF647 and ApoB‐CoraLite‐Plus‐488 antibodies on sample and PBS, represented as bivariate plots with a quad gate.
**Figure S4**: A) Bivariate plots of CD9‐AF647 and ExoBrite CTB or ExoBrite True EV Membrane stain for Vehicle, GW4869 and PBS+stain. B) Median size in nm of the different gatings shown in Figure 2H‐I. C) Bivariate plots of vSSC1‐H and vFSC‐H overlaid with the different ApoB± populations.

## Data Availability

The data that support the findings of this study are available from the corresponding author upon reasonable request.
