# Supplementary material for: GW4869 Depletes Macrophages and Increases Number of Extracellular Vesicles in Murine Peritoneal Cavity Fluid
Source: J Extracell Biol. 2026 Aug 28;5(9):e70169. doi: 10.1002/jex2.70169 (PMC13522827; doi:10.1002/jex2.70169)
Supplement: Supplementary file 1 [file JEX2-5-e70169-s001.pdf]

Figure S1

A

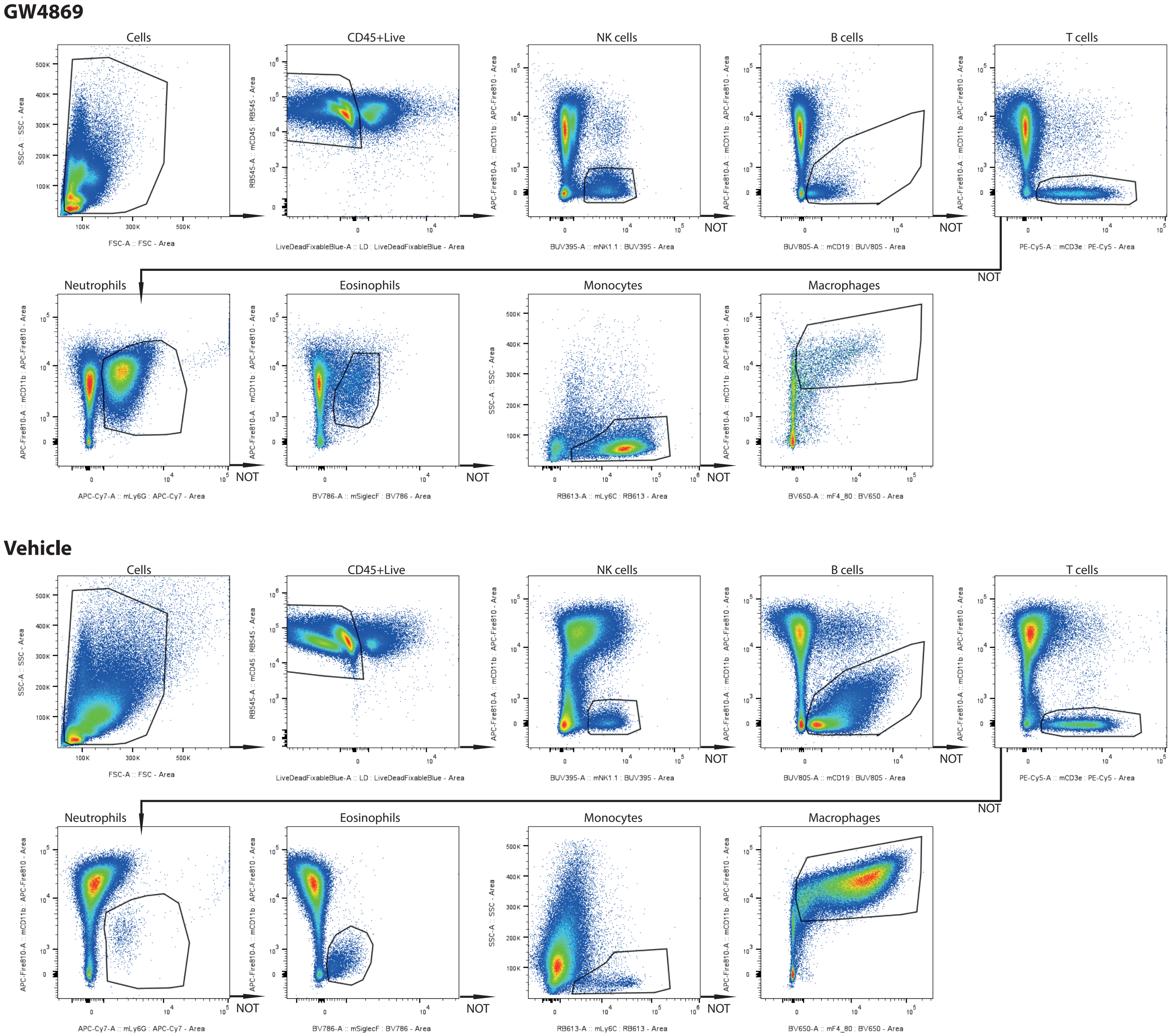
GW4869
Cells
CD45+Live
NK cells
B cells
T cells
NOT
Neutrophils
Eosinophils
Monocytes
Macrophages
Vehicle
SSC-A :: SSC - Area
FSC-A :: FSC - Area
RB545-A :: mCD45 : RB545 - Area
LiveDeadFixableBlue-A :: LD : LiveDeadFixableBlue - Area
APC-Fire810-A :: mCD11b : APC-Fire810 - Area
BUV395-A :: mNK1.1 : BUV395 - Area
BUV805-A :: mCD19 : BUV805 - Area
PE-Cy5-A :: mCD3e : PE-Cy5 - Area
APC-Cy7-A :: mLy6G : APC-Cy7 - Area
BV786-A :: mSiglecF : BV786 - Area
RB613-A :: mLy6C : RB613 - Area
BV650-A :: mF4_80 : BV650 - Area

B

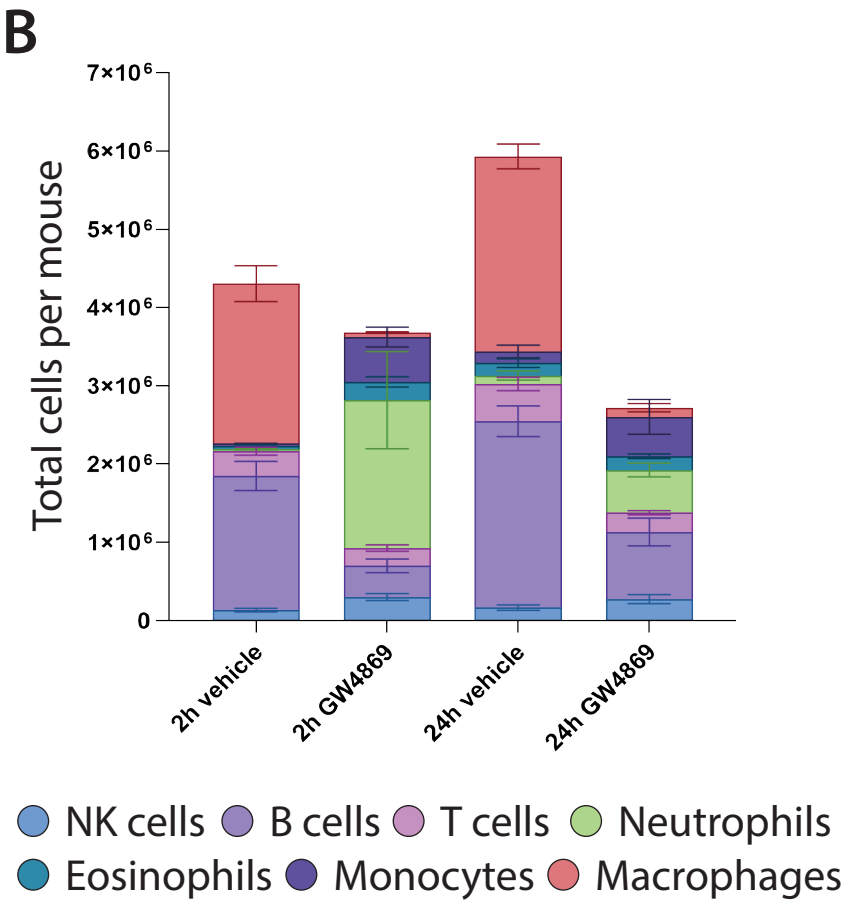
Total cells per mouse
7×10⁶
6×10⁶
5×10⁶
4×10⁶
3×10⁶
2×10⁶
1×10⁶
0
2h vehicle
2h GW4869
24h vehicle
24h GW4869
NK cells
B cells
T cells
Neutrophils
Eosinophils
Monocytes
Macrophages

Figure S2

A Inclusion gate --> Singlets

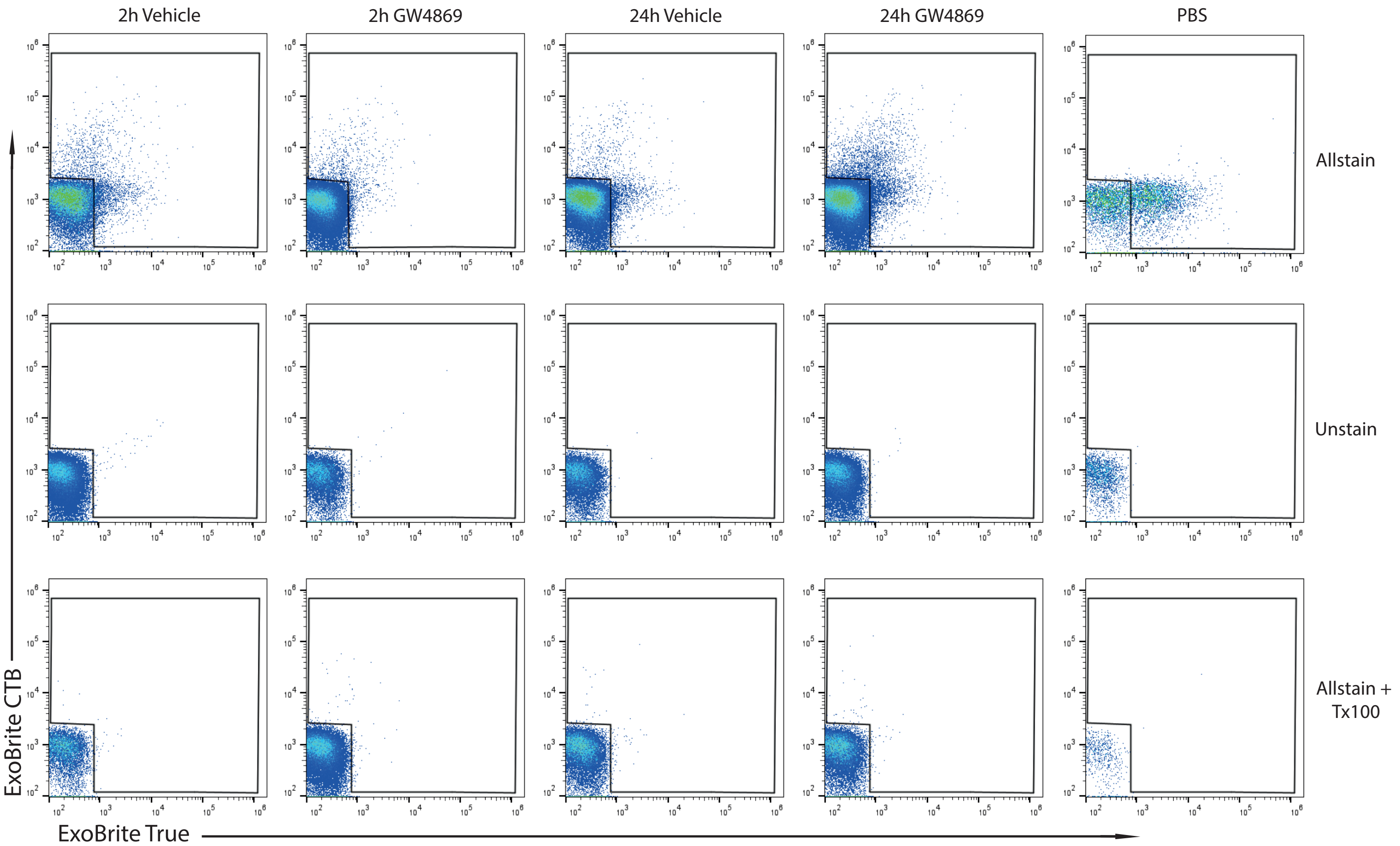

B Inclusion gate --> Singlets

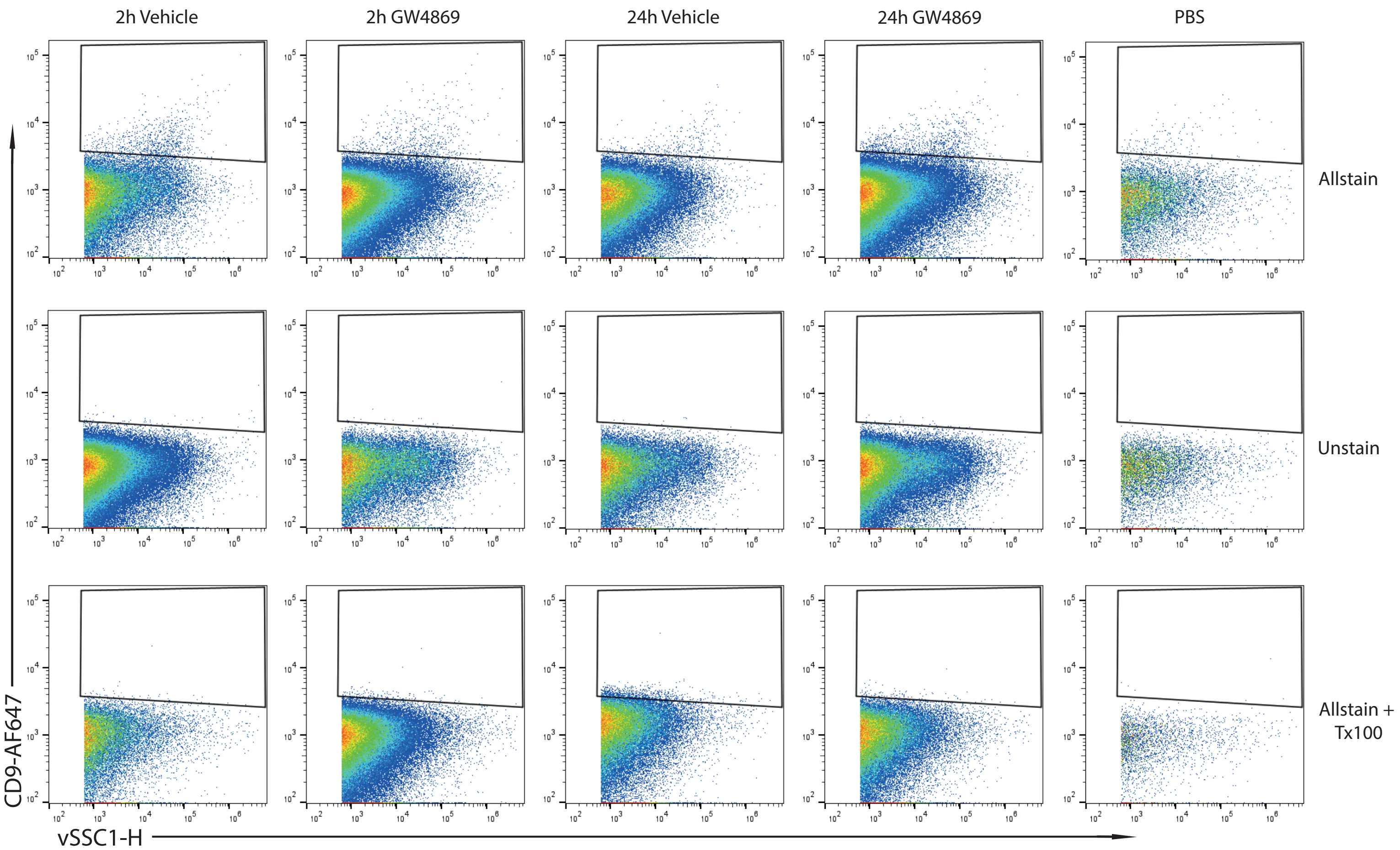

Figure S3

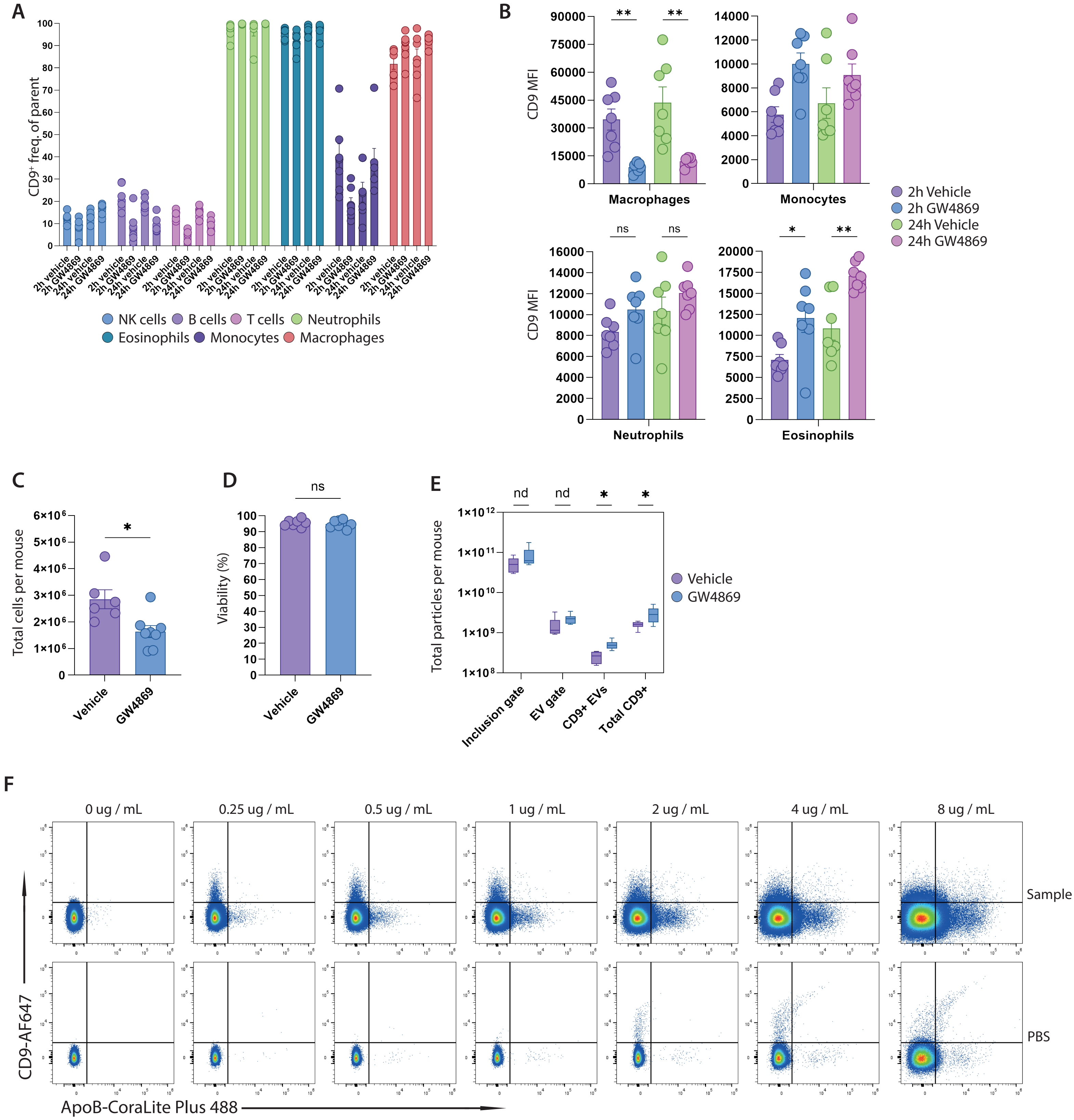

A
CD9+ freq. of parent
2h vehicle
2h GW4869
24h vehicle
24h GW4869
NK cells
B cells
T cells
Neutrophils
Eosinophils
Monocytes
Macrophages
B
CD9 MFI
Macrophages
Monocytes
Neutrophils
Eosinophils
2h Vehicle
2h GW4869
24h Vehicle
24h GW4869
C
Total cells per mouse
Vehicle
GW4869
D
Viability (%)
E
Total particles per mouse
Inclusion gate
EV gate
CD9+ EVs
Total CD9+
nd
ns
F
0 ug / mL
0.25 ug / mL
0.5 ug / mL
1 ug / mL
2 ug / mL
4 ug / mL
8 ug / mL
Sample
PBS
CD9-AF647
ApoB-CoraLite Plus 488

Figure S4

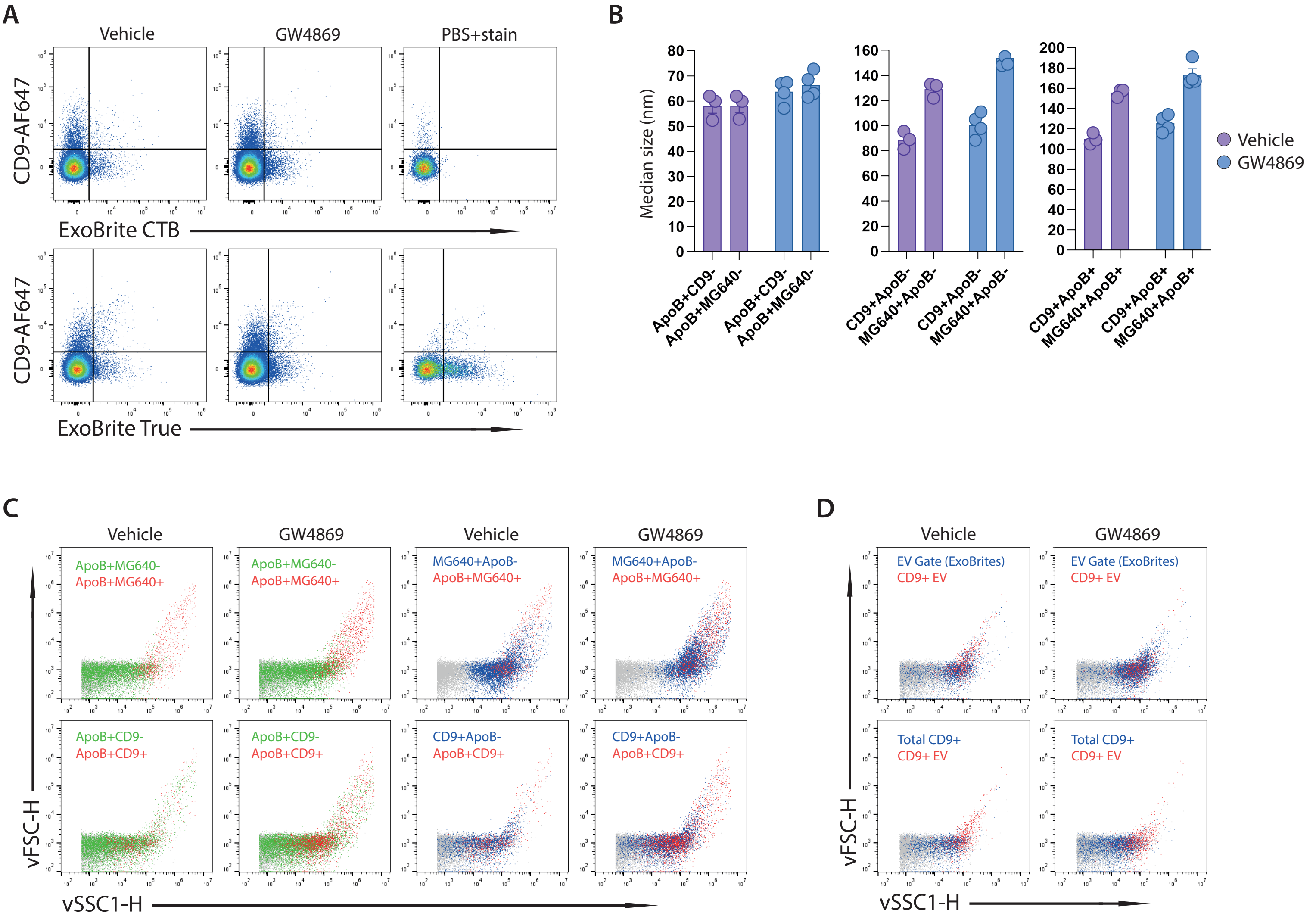

A
Vehicle
GW4869
PBS+stain
CD9-AF647
ExoBrite CTB
ExoBrite True
B
Median size (nm)
ApoB+CD9-
ApoB+MG640-
CD9+ApoB-
MG640+ApoB-
CD9+ApoB+
MG640+ApoB+
Vehicle
GW4869
C
Vehicle
GW4869
ApoB+MG640-
ApoB+MG640+
MG640+ApoB-
ApoB+CD9-
ApoB+CD9+
CD9+ApoB-
vFSC-H
vSSC1-H
D
EV Gate (ExoBrites)
CD9+ EV
Total CD9+
